# Efficacy of Electroacupuncture in the Treatment of Mild to Moderate Female Stress Urinary Incontinence: Protocol for a Systematic Review and Network Meta-Analysis

**DOI:** 10.2196/55870

**Published:** 2024-11-04

**Authors:** JiaNi Shi, Peiqi Li, Yifan Wu, Jiawei Li, Yuchen Zhang, Bin Xiao

**Affiliations:** 1 School of Acupuncture-Moxibustion and Tuina Shanghai University of Traditional Chinese Medicine Shanghai China

**Keywords:** stress urinary incontinence, electroacupuncture, drugs, pelvic floor muscle training, systematic review, network meta-analysis

## Abstract

**Background:**

Stress urinary incontinence (SUI), the most common form of urinary incontinence, is a condition that affects many women. It is characterized by involuntary urine leakage during activities that increase abdominal pressure, such as sneezing, coughing, or physical exertion, according to the International Continence Society. SUI affects patients’ quality of life and causes depression and emotional disorders, which negatively influences physical and mental health. The participants in the studies in this review comprised women with mild to moderate SUI, because there are more female patients than male patients, and most patients with severe SUI are treated surgically. Moreover, after retrieval, there were no systematic reviews or network meta-analyses (NMAs) of conservative treatments, such as electroacupuncture (EA), in women with mild to moderate SUI.

**Objective:**

This study aims to investigate the efficacy of electroacupuncture among women with mild to moderate SUI using an NMA.

**Methods:**

Randomized clinical trials related to conservative treatments for SUI will be searched in 5 English and 3 Chinese literature databases: EMBASE, PubMed, Cochrane, Web of Science, ClinicalTrials.gov, Chinese National Knowledge Infrastructure (CNKI), WanFang, and the Chinese BioMedical Literature Database. The search period for these 8 electronic databases will be from 2002 to 2022. The PROSPERO database and the International Platform of Registered Systematic Review and Meta-Analysis Protocols (INPLASY) database will also be searched. Two reviewers will independently complete the research selection. After screening the studies, 2 other researchers will extract the data, and the quality of the included studies will be evaluated according to the quality standards specified in the Cochrane Collaboration Tool (version 2). The primary outcomes will be the change in urine leakage determined by a 1-hour pad test and International Consultation on Incontinence Questionnaire Short Form (ICIQ-SF) scores at baseline and at the conclusion of the follow-up. The secondary outcomes will be 72-hour incontinence episodes, residual bladder volume, effective rate, urodynamic indexes, and other reported measurements. Stata (version 14.0; StataCorp) and Review Manager (RevMan version 5.3; Cochrane) will be implemented for data synthesis and meta-analysis.

**Results:**

The results are not yet accessible because this is a protocol for a systematic review and meta-analysis. The protocol was registered on INPLASY on February 22, 2023. By April 6, 2023, we had completed the literature search of the 8 databases and completed the selection and data extraction of the articles.

**Conclusions:**

The results of this systematic review will demonstrate the efficacy of EA among women with mild to moderate SUI. The results will provide evidence for clinicians and guideline makers to choose suitable treatments for SUI.

**Trial Registration:**

International Platform of Registered Systematic Review and Meta-Analysis Protocols (INPLASY) 202320098; https://inplasy.com/inplasy-2023-2-0098/

**International Registered Report Identifier (IRRID):**

DERR1-10.2196/55870

## Introduction

Stress urinary incontinence (SUI), the most common type of urinary incontinence (UI), is defined by the International Continence Society as an involuntary loss of urine on physical exertion, sneezing, or coughing [1]. The incidence of UI is 2-3 times higher in women than in men [2]. Epidemiological studies have demonstrated that SUI is a common health problem worldwide, affecting 23% to 45% of the female population [3]. The prevalence of SUI in China is 18.9% [4], with the highest prevalence (28%) in the age group of 50-59 years [5].

Although SUI is not a life-threatening disorder, it reduces quality of life in both the physical and mental health domains, and the condition is an independent risk factor for anxiety and depression, which may negatively impact work function [6]. According to urodynamic examination, involuntary urine leakage occurs when the bladder pressure exceeds the maximum urethral pressure, in the presence of increased abdominal pressure and no detrusor contraction. In the anatomical pathogenesis of SUI, the urethra itself, the periurethra, and the pelvic floor nerves all play significant roles [7]. Vaginal delivery is a significant risk factor for developing pelvic floor diseases such as SUI [8]. The levator ani muscle plays an important role in supporting the pelvic organs, and women are most likely to damage this muscle during pregnancy and childbirth because of overstretching. In addition to other anatomical factors, the external urethral sphincter and the levator ani muscle’s joint contraction play important roles in urinary continence, and they can result in the formation of a urethral bend angle and the urethra being forcibly closed. When the external urethral sphincter and levator ani muscle are dysfunctional, the urethra cannot be forcibly closed, which causes urinary incontinence [7].

SUI is divided into mild, moderate, and severe levels with the Ingelman-Sundberg scale. Mild UI occurs when coughing or sneezing, with no need to use a urinary pad; moderate UI occurs in daily activities such as running, jumping, and brisk walking and requires the use of a urinary pad; severe UI occurs with slight activity and the change to a supine position [9].

SUI, irrespective of its severity, has a great impact on patients’ quality of life. But due to the embarrassment of describing their symptoms, only the most severely affected women seek help [10]. In the past 20 years, scholars worldwide have conducted research on various aspects of SUI, encompassing its etiology, diagnostic methodologies, and therapeutic interventions. Chinese scholars have found 20 risk factors for SUI, including old age, high BMI, heavy labor intensity, a history of drinking alcohol, hypertension, constipation, a history of urinary system disease, a history of respiratory system disease, a history of gynecological disease, giving birth more than once, heavy birth weight of the first child, perineal laceration, a history of pelvic surgery, menopause, vaginal delivery, and uterine prolapse [11].This extensive list underscores the multifactorial nature of SUI and highlights the need for a comprehensive understanding of the condition to inform effective clinical management strategies.

The International Consultation on Urological Diseases recommends that treatment methods for mild and moderate SUI include lifestyle intervention, behavior therapy, and pelvic floor muscle training (PFMT). [12]. PFMT has been demonstrated to be effective in alleviating symptoms of SUI across a spectrum of age groups, including young, middle-aged, and older women who have either SUI or mixed urinary incontinence (MUI) [13].

Although PFMT has a significant impact on female SUI, traditional PFMT is based on one-on-one training sessions with pelvic floor physical therapists or other licensed health care specialists. This form of intense, individual rehabilitation therapy is expensive, and the community’s availability of licensed pelvic floor therapists is restricted [14].

In addition to conservative treatment, many women also opt for surgery. The standard surgery for SUI includes retro-pubic urethropexy or a pubovaginal sling. Mid-urethral slings are currently the most regularly performed surgical option [15]. Women younger than 60 years rate the outcome of mid-urethral sling surgery better than older women. And women aged 60 to 79 years have a slight but statistically significant increased risk of surgical complications, such as bladder perforation and urine retention, as well as an increase in urgent urinary incontinence (UUI) symptoms and de novo UUI [16]. Surgical treatment of female SUI is effective, but there is a risk of pain, infection, and dysuria-related complications [17]. As a result, many patients are hesitant to have surgery. Therefore, other safe and effective conservative therapies are needed.

There are clinical trials showing that electroacupuncture (EA) is more effective than PFMT in the treatment of SUI [18]. However, the efficacy of PFMT at home heavily depends on adherence and standard movements, whose quality is more difficult to control than EA.

Recently, many clinical studies and reports have revealed that the effectiveness of simple EA therapy or EA-related therapies for treating SUI is relatively high. A randomized clinical trial [19] showed that EA involving the lumbosacral region in women with SUI resulted in less urine leakage after 6 weeks compared to sham EA. In another trial [20], based on the International Consultation on Incontinence Questionnaire Short Form (ICIQ-SF) score, EA showed better results than sham EA in improving quality of life, indicating that EA had sound short-term and long-term specific effects. EA continued to work even after treatment was stopped.

Although current clinical trials have shown the effectiveness of EA, meta-analysis assessing its efficacy and safety is constrained by limitations inherent in previous clinical studies, such as small sample sizes, suboptimal study designs, and significant risk of bias [21]. Consequently, a rigorous scientific and systematic assessment of EA’s efficacy remains needed. This study aims to compare the efficacy of EA with other conservative therapies in treating female SUI of mild and moderate severity by using network meta-analysis (NMA), providing evidence for clinical decision-making.

## Methods

### Study Registration

This protocol was registered with the International Platform of Registered Systematic Review and Meta-Analysis Protocols (INPLASY). The registry number is INPLASY 202320098 [22], and any revisions to the program will be documented on the INPLASY platform.

### Search Strategy

We will electronically search (search period: 2002 to 2022) the following international and domestic databases: EMBASE, PubMed, Cochrane, Web of Science, ClinicalTrials.gov, Chinese National Knowledge Infrastructure (CNKI), WanFang, and the Chinese Biomedical Literature Database (CBM). The PROSPERO database and the INPLASY database will also be searched. The search strategy will use a combination of terms from Medical Subject Headings (MeSH) and keywords including *SUI*, *EA*, *drugs*, *PFMT*, *systematic review*, and *NMA* (Multimedia Appendix 1). The search words in the Chinese databases have the same meaning as those used in the English databases. Protocols and conference reports will be excluded. The document languages will be limited to Chinese and English.

### Criteria for Studies

#### Types of Studies

All randomized controlled trials (RCTs) will be included. Protocols, observational research, animal experiments, qualitative research, letters, news, communications, and review articles will not be included. Duplicate articles, irrelevant research, articles with missing data and/or methodology issues, and articles whose full text could not be found will be excluded.

#### Types of Participants

Female patients of any age or ethnicity with a clinical diagnosis of SUI in accordance with the diagnostic criteria of the International Continence Society [23] will be included. Postpartum female patients will be included. Male participants with SUI will be excluded. Patients who have undergone surgery will be excluded. Patients diagnosed with mild or moderate SUI will be included, while those with severe SUI and other types of UI, such as UUI and MUI, will be excluded. Patients who have undergone other previous treatments, such as surgical treatment and drug treatment, will be excluded.

#### Types of Interventions

The studies will be included if they use EA or transcutaneous electric nerve stimulation (TENS) as the treatment for SUI.

#### Types of Comparators and Controls

Studies will be included if they used medicine exclusively (for example, selective adrenoceptor agonists, midodrine hydrochloride, duloxetine hydrochloride, and topical vaginal estrogen therapy) or any PFMT exclusively. Studies will be excluded if they (1) do not incorporate a comparison group or condition or (2) use EA (including TENS) as an adjuvant therapy.

### Outcome Measures

#### Primary Outcome

The primary outcome is the change from baseline to the end of follow-up in urine leakage, measured by a 1-hour pad test and the ICIQ-SF score.

#### Secondary Outcomes

The secondary outcomes include 72-hour incontinence episodes, residual bladder volume, effective rate, urodynamic indexes, and other reported measurements with sufficient data for synthesis.

### Data Collection and Analysis

#### Selection of Studies

The search results from the above databases will be exported to EndNote (Clarivate) [24] for article screening. Duplicate articles will be filtered and removed. Two reviewers (JS and JL) will independently select articles by screening their titles and abstracts. Full texts will be searched for further evaluation when necessary. Then, the reviewers will examine the full-text articles according to the inclusion criteria. In cases of conflicting opinions, a third reviewer (BX) will be consulted to resolve any disagreement.

#### Data Extraction and Management

Three reviewers (PL, YZ, and YW) will independently extract parameters from functional studies, including identification information (eg, publication year and first author), general information (eg, country, number of centers, and sample size), baseline characteristics of the participants (eg, age, BMI, SUI severity, and mode of child delivery), interventions (type of acupuncture, as well as frequency, sessions, and duration), comparator (if there are any; this will include details of the treatment, such as its name, dosage, frequency, and course), and outcomes (data and time points for each measurement, safety of the treatments, and adverse effects).

#### Risk of Bias (Quality) Assessment

Two researchers (PL and YZ) will independently evaluate the literature’s quality, mainly according to results obtained with the Cochrane Risk of Bias 2 assessment tool [25]. The main factors considered include the randomization process, deviation from the intended intervention, missing outcome data, measurement of the outcome, selective outcome reporting, and other sources of bias. Two researchers will grade the above content as “low bias risk,” “high bias risk,” or “unclear bias risk” and cross-check the obtained results. Any conflicts or discrepancies will be solved by discussion; otherwise, a third researcher (BX) will be consulted to achieve agreement. Finally, a bias risk diagram will be drawn using Microsoft Excel 2019.

#### Quality of Evidence Assessment

Additionally, if possible, we will use the Grading of Recommendations Assessment, Development and Evaluation (GRADE) framework [26] to assess the certainty of evidence. We will assess the quality of the evidence in 4 levels: high quality, moderate quality, low quality, and very low quality. Moreover, the official GRADE Pro software will be used to conduct this process.

### Statistical Analysis

#### Overview

Review Manager (RevMan version 5.3; Cochrane) will be used to perform statistical analysis. A pairwise meta-analysis will be used to assess treatments against direct evidence. Considering the expected between-study heterogeneity, we will use a random effects (RE) model for each intervention comparison. We will pool the data from each outcome separately using a Bayesian RE model. A risk ratio with 95% CIs will be used to analyze dichotomous outcomes. The continuous data will be expressed as means and SDs for each study. The mean difference, or standardized mean difference if different metrics are used across studies, will be calculated with their respective 95% CIs. We will calculate the rate ratio with a 95% CI for count outcomes.

Heterogeneity will be evaluated with the *χ*^2^ test and the *I*^2^ statistic [25]. Statistical heterogeneity will be categorized as follows: (1) *P*>.05 and *I*^2^≤ 50% indicates low or moderate heterogeneity, and a fixed effect model can be used for the analysis; (2) *P*<.05 and *I*^2^=50%-75% indicates substantial heterogeneity; and (3) *P*<.05 and *I*^2^≥75% indicates considerable or high heterogeneity [27].

We will perform an NMA for each outcome to compare multiple interventions in a single model using Stata (version 14.0; StataCorp). We will preferentially pool direct evidence; however, indirect comparisons will be used to estimate the effect in the absence of direct comparisons. For analysis, the Markov chain Monte Carlo method can be used. In addition, we will evaluate convergence using Gelman-Rubin diagnostics and inspection of Monte Carlo errors [28].

The intervention effect estimates will be presented along with their corresponding 95% CIs, and surface under the cumulative ranking curve (SUCRA) will also be shown. The best treatment is expected to have high SUCRA values, while the worst will have low values. We will present the direct, indirect, and network estimates for each comparison.

#### Sensitivity Analysis

The effect of studies with a high risk of bias on the overall estimate of the intervention’s effect will be determined using sensitivity analysis.

#### Subgroup Analysis

To investigate sources of heterogeneity, we will conduct subgroup analyses according to the following factors: age, disease course, severity of SUI, acupoints, waveform, frequency of EA parameters, and treatment duration.

#### Dealing With Missing Data

Should clarification or additional data be necessary from the included articles, the corresponding authors of these studies will be contacted via telephone or email.

#### Assessment of Reporting Bias

If more than 10 studies are included, a funnel plot will be used to construct the reporting bias. Otherwise, we will use Stata 14 to assess reporting bias with the Egger test [29].

## Results

**Figure 1:**
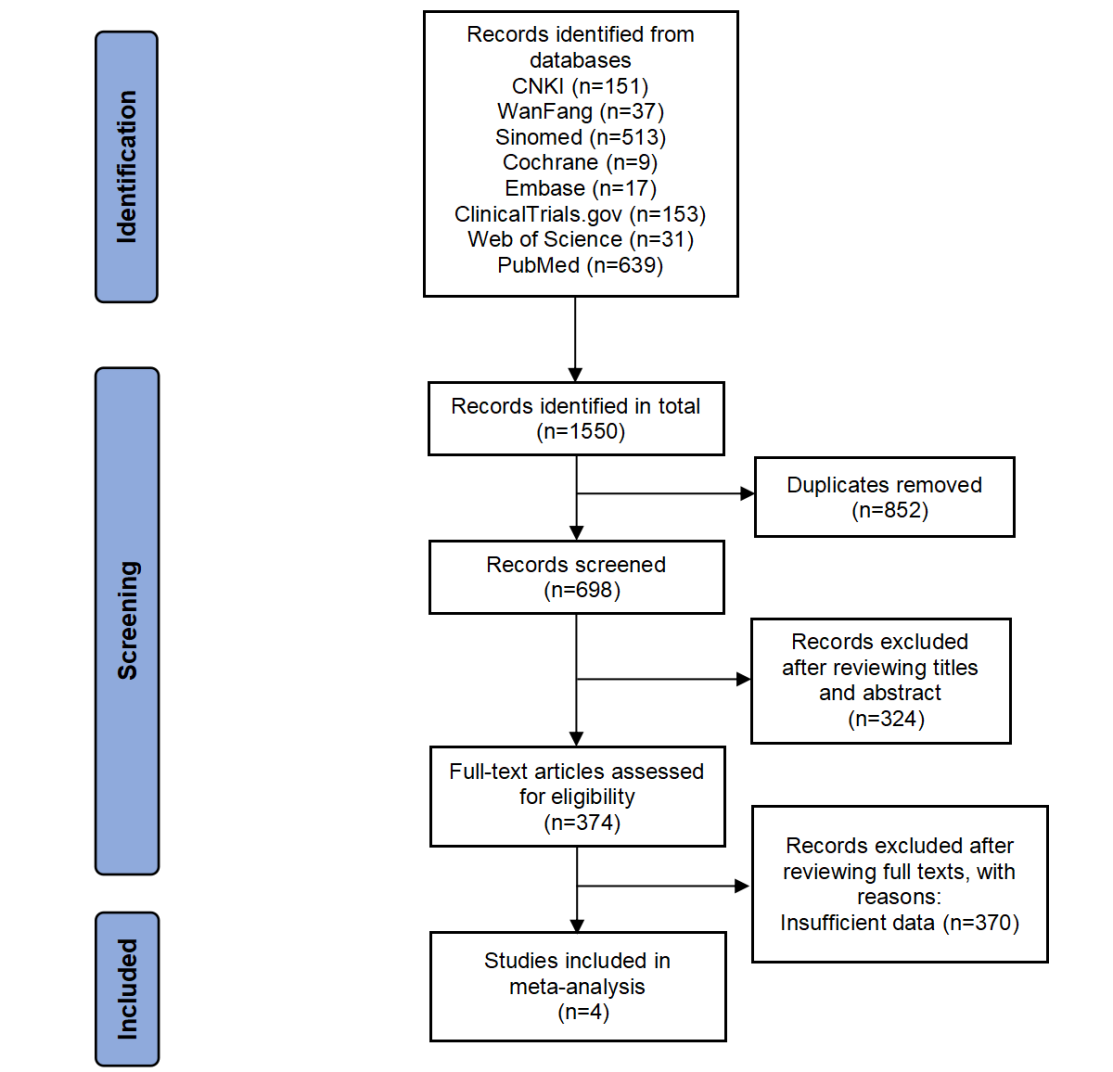
PRISMA (Preferred Reporting Items for Systematic reviews and Meta-Analyses) 2020 flow diagram for new systematic reviews, including searches of databases and registers only. CNKI: Chinese National Knowledge Infrastructure.

Results are not yet available because this is a protocol for a systematic review and meta-analysis. The protocol was registered on INPLASY on February 22, 2023. By April 6, 2023, we had completed the literature search of 8 databases and completed the selection and data extraction of the articles. The specific analysis has not been completed yet. The search and selection process is illustrated by the flow diagram in Figure 1.

## Discussion

### Principal Findings

SUI is a significant problem that seriously affects people’s health. The prevailing theory explaining SUI is that leakage from the urethra occurs when the intra-abdominal pressure exceeds the urethral pressure [30]. Intrinsic urethral sphincter deficiency and urethral hypermobility caused by weak pelvic floor muscles have been proposed as the main pathophysiologies [31].

EA is a treatment in which a small amount of low-frequency pulse current is applied by an acusector to a filiform needle inserted into an acupoint during acupuncture. Although the treatment mechanism of acupuncture for SUI is currently unknown, EA may facilitate the reinnervation and strengthening of the pelvic floor muscles, improving symptoms of SUI [19]. Moreover, the cost of acupuncture treatment is generally lower than that of Western medicine [32,33].

### Summary

To sum up, this review aims to determine whether EA is an effective, safe, and affordable treatment for women with mild to moderate SUI when compared with other conservative treatments, such as drug therapy and PFMT.

Previous research [3,19,34] has shown that EA is frequently used in the clinical treatment of SUI and is an efficient and safe treatment. Based on preliminary screening and data extraction results for the change in urine leakage from baseline to the end of the follow-up, as measured by a 1-hour pad test, the ICIQ-SF score, 72-hour incontinence episodes, and effective rate measurements, it appears that EA therapy is more effective in treating mild to moderate SUI in women compared to drug therapy or PFMT.

### Comparison to Prior Work

Most current meta-analyses have extensively searched and analyzed conservative treatment methods for female SUI, despite the low consistency and high heterogeneity among the included studies. Compared with other meta-analyses for conservative treatment of female SUI [34-37], we narrowed the range of interventions and limited the severity of female SUI to mild to moderate, making the analysis more targeted.

### Strengths and Limitations

This systematic review and meta-analysis of the research should provide an integrated understanding of the current state of treatment for mild to moderate SUI in women, offer more ideas to clinicians for treating the condition, and provide a basis for patients to choose more effective, safer, and more convenient and affordable treatment options. Our NMA will be the first to compare the efficacy of EA or TENS with drugs or PFMT in the treatment of women with mild to moderate SUI.

Nevertheless, there may also be some potential limitations in this review. Due to limited language ability, financial resources, and personnel, only papers written in English or Chinese will be included. This limitation may inadvertently restrict the breadth of available data or introduce a linguistic bias.

Since the quality of the original literature will affect the quality of this evaluation, we will include the literature in strict accordance with outcome indicators and screening criteria to reduce the impact of literature quality on the risk of bias.

### Future Directions

The findings of this systematic review will be used as evidence to determine if EA treatment can be used to intervene effectively in women with mild to moderate SUI. This NMA will also help clinicians make evidence-based clinical choices and provide guideline makers with high-quality updated evidence for recommending EA.

## Appendix

Multimedia Appendix 1 Full search strategies.

## Abbreviations

CBM: Chinese Biomedical Literature Database
CNKI: Chinese National Knowledge Infrastructure
EA: electroacupuncture
GRADE: Grading of Recommendations Assessment, Development and Evaluation
ICIQ-SF: International Consultation on Incontinence Questionnaire Short Form
INPLASY: International Platform of Registered Systematic Review and Meta-Analysis Protocols
MeSH: Medical Subject Headings
MUI: mixed urinary incontinence
NMA: network meta-analysis
PFMT: pelvic floor muscle training
RCT: randomized controlled trial
RE: random effects
SUCRA: surface under the cumulative ranking curve
SUI: stress urinary incontinence
TENS: transcutaneous electric nerve stimulation
UI: urinary incontinence
UUI: urgent urinary incontinence
