# Supplementary material for: Efficacy of Electroacupuncture in the Treatment of Mild to Moderate Female Stress Urinary Incontinence: Protocol for a Systematic Review and Network Meta-Analysis
Source: JMIR Res Protoc. 2024 Nov 4;13:e55870. doi: 10.2196/55870 (PMC11574501; doi:10.2196/55870)
Supplement: Multimedia Appendix 1 [file resprot_v13i1e55870_app1.pdf]

Multimedia Appendix 1: Full Search Strategies

Database	Search Strategy
**Pubmed**	1. "Urinary Incontinence, Stress"[Mesh]<br>2. ((("Urinary Incontinence, Stress"[Mesh]) OR (Urinary Stress Incontinence[Title/Abstract])) OR (Incontinence, Urinary Stress[Title/Abstract])) OR (Stress Incontinence, Urinary[Title/Abstract])<br>3. #1 OR #2<br>4. "Electroacupuncture"[Mesh]<br>5. ("Electroacupuncture"[Mesh]) OR (Electroacupuncture[Title/Abstract])<br>6. #4 OR #5<br>7. "Transcutaneous Electric Nerve Stimulation"[Mesh]<br>8. ((((((((((((((((((((((((((((("Transcutaneous Electric Nerve Stimulation"[Mesh]) OR (Electric Stimulation, Transcutaneous[Title/Abstract])) OR (Stimulation, Transcutaneous Electric[Title/Abstract])) OR (Transcutaneous Electric Stimulation[Title/Abstract])) OR (Percutaneous Electric Nerve Stimulation[Title/Abstract])) OR (Electrical Stimulation, Transcutaneous[Title/Abstract])) OR (Transcutaneous Electrical Stimulation[Title/Abstract])) OR (Transdermal Electrostimulation[Title/Abstract])) OR (Electrostimulation, Transdermal[Title/Abstract])) OR (Percutaneous Electrical Nerve Stimulation[Title/Abstract])) OR (Transcutaneous Electrical Nerve Stimulation[Title/Abstract])) OR (Transcutaneous Nerve Stimulation[Title/Abstract])) OR (Nerve Stimulation, Transcutaneous[Title/Abstract])) OR (Stimulation, Transcutaneous Nerve[Title/Abstract])) OR (TENS[Title/Abstract])) OR (Percutaneous Neuromodulation Therapy[Title/Abstract])) OR (Neuromodulation Therapy, Percutaneous[Title/Abstract])) OR (Percutaneous Neuromodulation Therapies[Title/Abstract])) OR (Therapy, Percutaneous Neuromodulation[Title/Abstract])) OR (Percutaneous Electrical Neuromodulation[Title/Abstract])) OR (Electrical Neuromodulation, Percutaneous[Title/Abstract])) OR (Electrical Neuromodulations, Percutaneous[Title/Abstract])) OR (Neuromodulation, Percutaneous Electrical[Title/Abstract])) OR (Neuromodulations, Percutaneous Electrical[Title/Abstract])) OR (Percutaneous Electrical Neuromodulations[Title/Abstract])) OR (Analgesic Cutaneous Electrostimulation[Title/Abstract])) OR (Cutaneous Electrostimulation, Analgesic[Title/Abstract])) OR (Electrostimulation, Analgesic Cutaneous[Title/Abstract])) OR (Electroanalgesia[Title/Abstract])) OR (Electroanalgesias[Title/Abstract])<br>9. #7 OR #8

	10. #6 OR #9<br>11. (((Pelvic floor physiotherapy[Title/Abstract]) OR (pelvic floor muscle training[Title/Abstract])) OR (pelvic floor exercise[Title/Abstract])) OR (Kegel exercise[Title/Abstract])<br>12. "Adrenergic Agonists"[Mesh]<br>13. ((((((((((("Adrenergic Agonists"[Mesh])) OR (Agonists, Adrenergic[Title/Abstract])) OR (Receptor Agonists, Adrenergic[Title/Abstract])) OR (Adrenomimetics[Title/Abstract])) OR (Adrenergic Agonist[Title/Abstract])) OR (Agonist, Adrenergic[Title/Abstract])) OR (Adrenergic Receptor Agonists[Title/Abstract])) OR (Agonists, Adrenergic Receptor[Title/Abstract])) OR (Adrenergic Receptor Agonist[Title/Abstract])) OR (Agonist, Adrenergic Receptor[Title/Abstract])) OR (Receptor Agonist, Adrenergic[Title/Abstract])<br>14. #12 OR #13<br>15. "Estrogens"[Mesh]<br>16. (((((((((((((("Estrogens"[Mesh]) OR (Estrogenic Compounds[Title/Abstract])) OR (Compounds, Estrogenic[Title/Abstract])) OR (Estrogenic Agents[Title/Abstract])) OR (Agents, Estrogenic[Title/Abstract])) OR (Estrogen[Title/Abstract])) OR (Estrogen Receptor Agonists[Title/Abstract])) OR (Agonists, Estrogen Receptor[Title/Abstract])) OR (Receptor Agonists, Estrogen[Title/Abstract])) OR (Estrogen Effect[Title/Abstract])) OR (Estrogenic Effect[Title/Abstract])) OR (Estrogenic Effects[Title/Abstract])) OR (Effects, Estrogenic[Title/Abstract])) OR (Estrogen Effects[Title/Abstract])) OR (Effects, Estrogen[Title/Abstract])<br>17. #15 OR #16<br>18. "Duloxetine Hydrochloride"[Mesh]<br>19. ((((((((((((((("Duloxetine Hydrochloride"[Mesh]) OR (Hydrochloride, Duloxetine[Title/Abstract])) OR (Duloxetine HCl[Title/Abstract])) OR (HCl, Duloxetine[Title/Abstract])) OR (LY 248686[Title/Abstract])) OR (LY-248686[Title/Abstract])) OR (LY248686[Title/Abstract])) OR (Duloxetine Ethanedioate (1:1), (+-)-isomer - T353987[Title/Abstract])) OR (LY 227942[Title/Abstract])) OR (LY-227942[Title/Abstract])) OR (LY227942[Title/Abstract])) OR (Duloxetine[Title/Abstract])) OR (N-methyl-3-(1-naphthalenyloxy)-3-(2-thiophene)propanamide[Title/Abstract])) OR (N-methyl-3-(1-naphthalenyloxy)-2-thiophenepropanamine[Title/Abstract])) OR (Duloxetine, (+)-isomer[Title/Abstract])) OR (Cymbalta[Title/Abstract])<br>20. #18 OR #19<br>21. #11 OR #14 OR #17 OR #20<br>22. #3 AND #10 AND #21

**Web of science**	(TS=(urinary incontinence, stress) OR AB=(Urinary Stress Incontinence OR Incontinence,Urinary Stress OR Stress Incontinence,Urinary)) AND (TS=(Electroacupuncture) OR TS=(Transcutaneous Electrical Nerve Stimulation) OR AB=(transcutaneous electric nerve stimulation OR Transcutaneous Electrical Nerve Stimulation OR Electric Stimulation, Transcutaneous OR Stimulation, Transcutaneous Electric OR Transcutaneous Electric Stimulation OR Percutaneous Electric Nerve Stimulation OR Electrical Stimulation, Transcutaneous OR Transcutaneous Electrical Stimulation OR Transdermal Electrostimulation OR Electrostimulation, Transdermal OR Percutaneous Electrical Nerve Stimulation OR Transcutaneous Electrical Nerve Stimulation OR Transcutaneous Nerve Stimulation OR Nerve Stimulation, Transcutaneous OR Stimulation, Transcutaneous Nerve OR TENS OR Percutaneous Neuromodulation Therapy OR Neuromodulation Therapy, Percutaneous OR Percutaneous Neuromodulation Therapies OR Therapy, Percutaneous Neuromodulation OR Percutaneous Electrical Neuromodulation OR Electrical Neuromodulation, Percutaneous OR Electrical Neuromodulations, Percutaneous OR Neuromodulation, Percutaneous Electrical OR Neuromodulations, Percutaneous Electrical OR Percutaneous Electrical Neuromodulations OR Analgesic Cutaneous Electrostimulation OR Cutaneous Electrostimulation, Analgesic OR Electrostimulation, Analgesic Cutaneous OR Electroanalgesia OR Electroanalgesias))AND (TS=(Pelvic floor muscle training) OR AB=(Pelvic floor physiotherapy OR pelvic floor muscle training OR pelvic floor exercise OR Kegel exercise) OR TS=(Adrenergic agonists) OR AB=(adrenergic agonists OR Agonists, Adrenergic OR Adrenergic Receptor Agonist OR Agonist, Adrenergic Receptor OR Receptor Agonist, Adrenergic OR Adrenergic Receptor Agonists OR Agonists, Adrenergic Receptor OR Receptor Agonists, Adrenergic OR Adrenergic Agonist OR Agonist, Adrenergic OR Adrenomimetics)OR TS=(estrogens) OR AB=(Oestrogen OR Estrogenic Compounds OR Compounds, Estrogenic OR Estrogenic Agents OR Agents, Estrogenic OR Estrogen OR Estrogen Receptor Agonists OR Agonists, Estrogen Receptor OR Receptor Agonists, Estrogen OR Estrogen Effect OR Estrogenic Effect OR Estrogenic Effects OR Effects, Estrogenic OR Estrogen Effects OR Effects, Estrogen)OR TS=(duloxetine hydrochloride) OR AB=(Duloxetine OR Hydrochloride, Duloxetine OR Duloxetine HCl OR HCl, Duloxetine OR LY 248686 OR LY-248686 OR LY248686 OR Duloxetine Ethanedioate (1:1), (+-)-isomer - T353987 OR LY 227942 OR LY-227942 OR LY227942 OR Duloxetine OR N-methyl-3-(1-naphthalenyloxy)-3-(2-thiophene)propanamide OR N-methyl-3-(1-naphthalenyloxy)-2-thiophenepropanamine OR Duloxetine, (+)-isomer OR Cymbalta)) NOT (TS=(animal

	experimentation) OR AB=(animal experiment OR Experimentation, Animal OR Animal Research OR Research, Animal OR Animal Experimental Use OR Animal Experimental Uses OR Experimental Use, Animal OR Experimental Uses, Animal OR Animal Experiments OR Animal Experiment OR Experiment, Animal OR Experiments, Animal OR guinea-pig OR rat OR rats OR rabbit))
**Cochrane library**	Search Name:<br>Date Run: 07/07/2024 16:13:00<br>Comment:<br><br>ID Search Hits<br>#1 MeSH descriptor: [Urinary Incontinence, Stress] explode all trees 1565<br>#2 Stress Incontinence, Urinary 3774<br>#3 Incontinence, Urinary Stress 3774<br>#4 Urinary Stress Incontinence 3774<br>#5 #1 or #2 or #3 or #4 3774<br>#6 MeSH descriptor: [Electroacupuncture] explode all trees 1172<br>#7 MeSH descriptor: [Transcutaneous Electric Nerve Stimulation] explode all trees 2854<br>#8 Electroanalgesias 0<br>#9 Electroanalgesia 65<br>#10 Analgesic Cutaneous Electrostimulation 20<br>#11 Cutaneous Electrostimulation, Analgesic 20<br>#12 Electrostimulation, Analgesic Cutaneous 20<br>#13 Electrostimulation, Analgesic Cutaneous 20<br>#14 Electrostimulation, Analgesic Cutaneous 20<br>#15 Percutaneous Electrical Neuromodulations 1<br>#16 Neuromodulation Therapy, Percutaneous 143<br>#17 Neuromodulation, Percutaneous Electrical 90<br>#18 Neuromodulations, Percutaneous Electrical 1<br>#19 Percutaneous Neuromodulation Therapies 43

	#20 Percutaneous Neuromodulation Therapies 43<br>#21 Percutaneous Neuromodulation Therapies 43<br>#22 Electrical Neuromodulations, Percutaneous 1<br>#23 Transcutaneous Nerve Stimulation 5019<br>#24 Percutaneous Electrical Nerve Stimulation 295<br>#25 Electrical Stimulation, Transcutaneous 4423<br>#26 Electric Stimulation, Transcutaneous 2496<br>#27 TENS 3223<br>#28 Percutaneous Electric Nerve Stimulation 205<br>#29 Transcutaneous Electrical Nerve Stimulation 3679<br>#30 Transcutaneous Electrical Nerve Stimulation 3679<br>#31 Nerve Stimulation, Transcutaneous 5019<br>#32 Stimulation, Transcutaneous Electric 2496<br>#33 Stimulation, Transcutaneous Nerve 5019<br>#34 Transdermal Electrostimulation 18<br>#35 Electrostimulation, Transdermal 18<br>#36 Transcutaneous Electrical Stimulation 4423<br>#37 #6 or #7 or #8 or #9 or #10 or #11 or #12 or #13 or #14 or #15 or #16 or #17 or #18 or #19 or #20 or #21 or #22 or #23 or #23 or #24 or #25 or #26 or #27 or #28 or #29 or #30 or #31 or #32 or #33 or #34 or #35 or #36 8089<br>#38 Pelvic floor physiotherapy 720<br>#39 pelvic floor muscle training 2155<br>#40 pelvic floor exercise 1764<br>#41 Kegel exercise 211<br>#42 #38 or #39 or #40 or #41 3079<br>#43 MeSH descriptor: [Adrenergic Agonists] explode all trees 4030<br>#44 Agonists16423<br>#45 Adrenergic 19162<br>#46 Receptor Agonists, Adrenergic 2015<br>#47 Agonist, Adrenergic 3221<br>#48 Adrenergic Receptor Agonists 2015<br>#49 Adrenomimetics 3<br>#50 Agonist, Adrenergic Receptor 1602<br>#51 Receptor Agonist, Adrenergic 1602<br>#52 Agonists, Adrenergic Receptor 2015<br>#53 Adrenergic Receptor Agonist 1602<br>#54 Adrenergic Agonist 3221<br>#55 #43 or #44 or #45 or #46 or #47 or #48 or #49 or #50 or #51 or #52 or #53 or #54 30815<br>#56 MeSH descriptor: [Estrogens] explode all trees 2371<br>#57 Estrogen Receptor Agonists 146<br>#58 Estrogen 15373<br>#59 Compounds, Estrogenic 59

	#60 Agonists, Estrogen Receptor; Agents, Estrogenic 11<br>#61 Receptor Agonists, Estrogen 146<br>#62 Estrogenic Agents 132<br>#63 Estrogenic Compounds 59<br>#64 Estrogen Effects 8403<br>#65 Effects, Estrogen 8403<br>#66 Estrogenic Effect 423<br>#67 Estrogen Effect 6766<br>#68 Estrogenic Effects 532<br>#69 Effects, Estrogenic 532<br>#70 #56 or #57 or #58 or #59 or #60 or #61 or #62 or #63 or #64 or #65 or #66 or #67 or #68 or #69 16249<br>#71 MeSH descriptor: [Duloxetine Hydrochloride] explode all trees 769<br>#72 Duloxetine, isomer 1<br>#73 LY 227942 0<br>#74 LY-227942 0<br>#75 LY227942 0<br>#76 Duloxetine 1947<br>#77 Cymbalta 84<br>#78 LY-248686 0<br>#79 LY248686 26<br>#80 LY 248686 0<br>#81 Hydrochloride, Duloxetine 858<br>#82 HCl, Duloxetine 10<br>#83 Duloxetine HCl 10<br>#84 #71 or #72 or #73 or #74 or #75 or #76 or #77 or #78 or #79 or #80 or #81 or #82 or #83 1954<br>#85 #42 or #55 or #70 or #84 51440<br>#86 #5 and #37 and #85 with Cochrane Library publication date Between Jan 2002 and Dec 2022, in Trials 9

**Clinical Trials.gov**	urinary incontinence, stress OR Urinary Stress Incontinence OR Incontinence, Urinary Stress OR Stress Incontinence, Urinary | electroacupuncture OR electroacupuncture OR (Pelvic floor physiotherapy) OR (pelvic floor muscle training) OR (pelvic floor exercise) OR (Kegel exercise) OR transcutaneous electric nerve stimulation OR Transcutaneous Electrical Nerve Stimulation OR Electric Stimulation, Transcutaneous OR Stimulation, Transcutaneous Electric OR Transcutaneous Electric Stimulation OR Percutaneous Electric Nerve Stimulation OR Transdermal Electrostimulation OR Electrostimulation, Transdermal OR Transcutaneous Electrical Nerve Stimulation OR Transcutaneous Nerve Stimulation OR Nerve Stimulation, Transcutaneous OR Stimulation, Transcutaneous Nerve OR TENS OR Percutaneous Neuromodulation Therapy OR Neuromodulation Therapy, Percutaneous OR Percutaneous Neuromodulation Therapies OR Therapy, Percutaneous Neuromodulation OR PercutaneousElectrical Neuromodulation OR Electrical Neuromodulation, Percutaneous OR Neuromodulation,Percutaneous Electrical OR Analgesic Cutaneous Electrostimulation OR Cutaneous Electrostimulation, Analgesic OR Electrostimulation, Analgesic Cutaneous OR Electroanalgesia OR Electroanalgesias OR Medicine OR adrenergic agonists OR adrenergic agonists OR Adrenergic agonists OR Agonists, Adrenergic OR Adrenergic Receptor Agonist OR Adrenergic Agonist OR Agonist, Adrenergic OR Adrenomimetics OR estrogens OR estrogens OR Oestrogen OR Estrogenic Compounds OR Estrogenic Agents OR Estrogen OR Estrogen ReceptorAgonists OR Agonists, Estrogen Receptor OR Receptor Agonists, Estrogen OR Estrogen Effect OR Estrogenic Effect OR duloxetine hydrochloride OR Duloxetine OR Hydrochloride, Duloxetine OR Duloxetine HCl OR HCl, Duloxetine OR LY 248686 OR Duloxetine Ethanedioate (1:1), (+-)-isomer - T353987 OR LY 227942 OR Duloxetine OR N-methyl-3-(1-naphthalenyloxy)-2-thiophenepropanamine OR Duloxetine, (+)-isomer OR Cymbalta | Study start from 01/01/2002 to 08/31/2022

**Embase**	Session Results<br>...<br>No. Query Results<br>Results Date<br>#19. #18 AND (2007:py OR 2009:py OR 2010:py OR 2013:py 17 9 Jul 2024<br>OR 2015:py OR 2016:py OR 2019:py OR 2020:py OR 2021:py) AND ('clinical article'/de OR 'clinical trial'/de OR 'comparative study'/de OR 'double blind procedure'/de OR 'human'/de OR 'human experiment'/de OR 'major clinical study'/de OR 'multicenter study'/de OR 'normal human'/de OR 'randomized controlled trial'/de OR 'randomized controlled trial topic'/de) AND [female]/lim<br>#18. #15 AND #16 AND #17 45 9 Jul 2024<br>#17. #7 OR #8 OR #9 OR #10 OR #11 OR #12 OR #13 OR #14 1,186,075 9 Jul 2024<br>#16. #3 OR #4 OR #5 OR #6 22,852 9 Jul 2024<br>#15. #1 OR #2 30,834 9 Jul 2024<br>#14. (((((('1 naphthyloxy':ti,ab,kw AND 3:ti,ab,kw 5,480 9 Jul 2024<br>AND '2 thienyl':ti,ab,kw AND 'n methylpropylamine':ti,ab,kw OR 3:ti,ab,kw) AND 'naphth 1 yloxy':ti,ab,kw AND 3:ti,ab,kw AND 'thien 2 yl':ti,ab,kw AND 'n methylpropylamine':ti,ab,kw OR 'ariclaim':ti,ab,kw OR 'cymbalta':ti,ab,kw OR 'dlx iso3':ti,ab,kw OR 'dlxiso3':ti,ab,kw OR 'drizalma':ti,ab,kw OR 'drizalma sprinkle':ti,ab,kw OR 'dulane':ti,ab,kw OR 'duloxetine boehringer ingelheim':ti,ab,kw OR 'duloxetine hydrochloride':ti,ab,kw OR 'duzela':ti,ab,kw OR 'ly 248686':ti,ab,kw OR 'ly248686':ti,ab,kw OR 'n methyl 3':ti,ab,kw) AND '1 naphthalenyloxy':ti,ab,kw AND '2 thiophenepropanamine':ti,ab,kw OR 'n methyl 3':ti,ab,kw) AND '1 naphthalenyloxy':ti,ab,kw AND 3:ti,ab,kw AND '2 thiophenyl':ti,ab,kw AND '1 propanamine':ti,ab,kw OR 'n methyl 3':ti,ab,kw) AND '1 naphthyloxy':ti,ab,kw AND 3:ti,ab,kw AND '2 thienyl':ti,ab,kw AND propylamine:ti,ab,kw OR

	'n methyl 3':ti,ab,kw) AND 'naphth 1 yloxy':ti,ab,kw AND 3:ti,ab,kw AND 'thien 2 yl':ti,ab,kw AND propylamine:ti,ab,kw OR 'n methyl 3':ti,ab,kw) AND 'naphthalen 1 yloxy':ti,ab,kw AND '2 thiophenepropanamine':ti,ab,kw OR 'n methyl 3':ti,ab,kw) AND 'naphthalen 1 yloxy':ti,ab,kw AND 3:ti,ab,kw AND 'thiophen 2 yl':ti,ab,kw AND 'propan 1 amine':ti,ab,kw OR 'nodetrip':ti,ab,kw OR 'xeristar':ti,ab,kw OR 'yentreve':ti,ab,kw OR 'duloxetine':ti,ab,kw<br>#13. 'duloxetine'/exp<br>15,162 9 Jul 2024<br>#12. 'alpha estrogen':ti,ab,kw OR 'alpha<br>231,586 9 Jul 2024<br>oestrogen':ti,ab,kw OR 'beta estrogen':ti,ab,kw OR 'beta oestrogen':ti,ab,kw OR 'estrogen uptake':ti,ab,kw OR 'estrogene':ti,ab,kw OR 'estrogenic agent':ti,ab,kw OR 'estrogenic hormone':ti,ab,kw OR 'estrogenic steroids, alkylated':ti,ab,kw OR 'estrogens':ti,ab,kw OR 'estrogens, non steroidal':ti,ab,kw OR 'estrogens, non-steroidal':ti,ab,kw OR 'kober chromogen':ti,ab,kw OR 'oestrogen':ti,ab,kw OR 'oestrogen uptake':ti,ab,kw OR 'oestrogene':ti,ab,kw OR 'oestrogenic agent':ti,ab,kw OR 'oestrogenic hormone':ti,ab,kw OR 'oestrogenic steroids, alkylated':ti,ab,kw OR 'oestrogens':ti,ab,kw OR 'oestrogens, non steroidal':ti,ab,kw OR 'oestrogens, non-steroidal':ti,ab,kw OR 'estrogen':ti,ab,kw<br>#11. 'estrogen'/exp<br>337,010 9 Jul 2024<br>#10. 'adrenergic agent':ti,ab,kw OR 'adrenergic 31,704<br>9 Jul 2024<br>agents':ti,ab,kw OR 'adrenergic agonist':ti,ab,kw OR 'adrenergic agonists':ti,ab,kw OR 'adrenergic drug':ti,ab,kw OR 'adrenergic receptor agonist':ti,ab,kw OR 'adrenergic receptor agonists':ti,ab,kw OR 'adrenergic receptor stimulant':ti,ab,kw OR 'adrenergic receptor stimulator':ti,ab,kw OR 'adrenergic stimulant':ti,ab,kw OR 'adrenergic stimulating agent':ti,ab,kw OR 'adrenergic

	stimulator':ti,ab,kw OR 'adrenoceptor agonist':ti,ab,kw OR 'adrenoceptor stimulating agent':ti,ab,kw OR 'adrenoceptor stimulator':ti,ab,kw OR 'adrenogenic agent':ti,ab,kw OR 'adrenomimetic agent':ti,ab,kw OR 'aminergic agent':ti,ab,kw OR 'aminergic drug':ti,ab,kw OR 'sympathetic drug':ti,ab,kw OR 'sympathicomimetic':ti,ab,kw OR 'sympathicomimetic agent':ti,ab,kw OR 'sympathicotropic agent':ti,ab,kw OR 'sympathomimetic':ti,ab,kw OR 'sympathomimetic agent':ti,ab,kw OR 'sympathomimetic drug':ti,ab,kw OR 'sympathomimetics':ti,ab,kw OR 'adrenergic receptor stimulating agent':ti,ab,kw<br>#9. 'adrenergic receptor stimulating agent'/exp 741,217 9 Jul 2024<br>#8. 'kegel exercise':ti,ab,kw OR 'kegel 4,110 9 Jul 2024<br>exercises':ti,ab,kw OR 'pelvic floor exercise':ti,ab,kw OR 'pelvic floor exercises':ti,ab,kw OR 'pelvic floor muscle exercise':ti,ab,kw OR 'pelvic floor muscle exercises':ti,ab,kw OR 'pelvic floor training':ti,ab,kw OR 'pelvic muscle exercise':ti,ab,kw OR 'pelvic muscle exercises':ti,ab,kw OR 'pelvic muscle training':ti,ab,kw OR 'pelvic floor muscle training':ti,ab,kw<br>#7. 'pelvic floor muscle training'/exp 4,815 9 Jul 2024<br>#6. ('electrostimulation, transcutaneous':ti,ab,kw OR 5,210 9 Jul 2024<br>'nerve stimulation, transcutaneous':ti,ab,kw OR 'percutaneous electric nerve stimulation':ti,ab,kw OR 'percutaneous electrical nerve stimulation':ti,ab,kw OR tens:ti,ab,kw) AND 'transcutaneous electrical nerve stimulation':ti,ab,kw OR 'transcutaneous electric nerve stimulation':ti,ab,kw OR 'transcutaneous electrical stimulation':ti,ab,kw OR 'transcutaneous electrostimulation':ti,ab,kw OR 'transcutaneous nerve stimulation':ti,ab,kw OR 'transcutaneous electrical nerve stimulation':ti,ab,kw

	#5. 'transcutaneous electrical nerve stimulation'/exp 10,559 9 Jul 2024<br>#4. 'acupuncture, electric':ti,ab,kw OR 'electric 9,656 9 Jul 2024<br>acupuncture':ti,ab,kw OR 'electrical acupoint stimulation':ti,ab,kw OR 'electrical acupuncture':ti,ab,kw OR 'electro-acupuncture':ti,ab,kw OR 'electrode acupuncture':ti,ab,kw OR 'electronic acupuncture':ti,ab,kw OR 'electroacupuncture':ti,ab,kw<br>#3. 'electroacupuncture'/exp 10,045 9 Jul 2024<br>#2. 'stress incontinence'/exp 27,703 9 Jul 2024<br>#1. 'incontinence, stress':ti,ab,kw OR 'stress 24,215 9 Jul 2024<br>urinary incontinence':ti,ab,kw OR 'stress urine incontinence':ti,ab,kw OR 'urinary incontinence, stress':ti,ab,kw OR 'urinary stress incontinence':ti,ab,kw OR 'urine stress incontinence':ti,ab,kw OR 'stress incontinence':ti,ab,kw
**Wanfang**	(Topic: (Stress Incontinence or female stress incontinence or mild stress incontinence or moderate stress incontinence or (SUI) or (Urinary Stress Incontinence)) AND topic: (electroacupuncture or transcutaneous acupoint electrical stimulation or (electroacupuncture)) AND Topics: (pelvic floor muscle training) or (pelvic floor exercise) or (Kegel exercise) or (Adrenergic agonists) or. (Pelvic floor muscle training) or (pelvic floor exercise) or (Kegel exercise) or (Adrenergic agonists) or Adrenergic Receptor Agonists or (Adrenomimetics) or (Oestrogen) or (Estrogenic Effect) (Duloxetine or duloxetine or (LY 248686) or (LY 227942) or (Cymbalta))) and Published time :2002-2022
**Sinomed**	(Stress Incontinence OR female stress incontinence OR mild stress incontinence OR SUI OR Urinary Stress Incontinence) AND (electroacupuncture OR percutaneous acupoint electrical stimulation OR electroacupuncture) AND (pelvic floor muscle training OR pelvic floor exercise OR Kegel exercise OR Adrenergic agonists OR Adrenomimetics OR Oestrogen OR Estrogenic. Adrenergic Receptor Agonists OR Adrenomimetics OR Oestrogen OR estrogenic Effect OR Duloxetine OR LY 248686 OR LY 227942 OR Cymbalta) Effect OR Duloxetine or ly 227942 or Cymbalta)

**CNKI**	SU %= (' Stress Incontinence' + 'female stress incontinence' + 'mild stress incontinence' + 'moderate stress incontinence' + 'SUI' + 'Urinary stress incontinence ') AND SU %= (' electroacupuncture' + 'transcutaneous acupuncture' + 'electroacupuncture') AND SU %= (' pelvic floor muscle training' + 'pelvic floor exercise' + 'Kegel exercise' + 'Adrenergic agonists' + 'Adrenomimetics' + Adrenergic Receptor Agonists' + 'Adrenomimetics' + 'Oestrogen' + 'estrogen' + 'Estrogenic Effect' + 'Duloxetine' + 'LY 248686' + 'LY 227942' + 'Cymbalta') AND YE BETWEEN ('2002', '2022')
